# Bioimpedance-assessed muscle wasting and its relation to nutritional intake during the first week of ICU: a pre-planned secondary analysis of Nutriti Study

**DOI:** 10.1186/s13613-024-01262-w

**Published:** 2024-02-17

**Authors:** Cristian Deana, Jan Gunst, Silvia De Rosa, Michele Umbrello, Matteo Danielis, Daniele Guerino Biasucci, Tommaso Piani, Antonella Cotoia, Alessio Molfino, Luigi Vetrugno, Nutriti Study Group

**Affiliations:** 1 Anesthesia and Intensive Care 1, Department of Anesthesia and Intensive Care, Health Integrated Agency of Friuli Centrale, Piazzale S. M. Della Misericordia 15, 33100 Udine, Italy; 2 Laboratory of Intensive-Care Medicine, Department of Cellular and Molecular Medicine, Louvain, Belgium; 3 Department of Intensive Care Medicine, University Hospitals Leuven, Louvain, Belgium; 4 Centre for Medical Sciences - CISMed, University of Trento, Trento, Italy; 5 Anesthesia and Intensive Care, Santa Chiara Regional Hospital, APSS Trento, Trento, Italy; 6 Department of Intensive Care and Anaesthesia, ASST Ovest Milanese, Legnano Hospital, Milan, Italy; 7 Laboratory of Studies and Evidence Based Nursing, Department of Cardiac, Vascular Sciences and Public Health, University of Padua, ThoracicPadua, Italy; 8 Department of Clinical Science and Translational Medicine, ‘Tor Vergata’ University of Rome, Rome, Italy; 9 Health Professions Staff, Health Integrated Agency of Friuli Centrale, Udine, Italy; 10 Department of Medical and Surgical Sciences, Anesthesia and Intensive Care Unit, Policlinico Riuniti Foggia, University of Foggia, Foggia, Italy; 11 Department of Translational and Precision Medicine, Sapienza University of Rome, Rome, Italy; 12 Department of Medical, Oral and Biotechnological Sciences, University of Chieti-Pescara, Chieti, Italy; 13 Department of Anesthesiology, Critical Care Medicine and Emergency, SS. Annunziata Hospital, Chieti, Italy

**Keywords:** Critically ill patient, Nutrition, Muscle wasting, ICU acquired weakness, Bioimpedance analysis, Proteins, Caloric debt

## Abstract

**Background:**

Muscle mass evaluation in ICU is crucial since its loss is related with long term complications, including physical impairment. However, quantifying muscle wasting with available bedside tools (ultrasound and bioimpedance analysis) must be more primarily understood. Bioimpedance analysis (BIA) provides estimates of muscle mass and phase angle (PA).

The primary aim of this study was to evaluate muscle mass changes with bioimpedance analysis during the first 7 days after ICU admission. Secondary aims searched for correlations between muscular loss and caloric and protein debt.

**Methods:**

Patients with an expected ICU-stay ≥ 72 h and the need for artificial nutritional support were evaluated for study inclusion. BIA evaluation of muscle mass and phase angle were performed at ICU admission and after 7 days. Considering the difference between ideal caloric and protein targets, with adequate nutritional macronutrients delivered, we calculated the caloric and protein debt. We analyzed the potential correlation between caloric and protein debt and changes in muscle mass and phase angle.

**Results:**

72 patients from September 1st to October 30th, 2019 and from August 1st to October 30th, 2021 were included in the final statistical analysis. Median age was 68 [59–77] years, mainly men (72%) admitted due to respiratory failure (25%), and requiring invasive mechanical ventilation for 7 [4–10] days. Median ICU stay was 8 [6–12] days. Bioimpedance data at ICU admission and after 7 days showed that MM and PA resulted significantly reduced after 7 days of critically illness, 34.3 kg vs 30.6 kg (p < 0.0001) and 4.90° vs 4.35° (p = 0.0004) respectively. Mean muscle loss was 3.84 ± 6.7 kg, accounting for 8.4% [1–14] MM reduction. Correlation between caloric debt (r = 0.14, p = 0.13) and protein debt (r = 0.18, p = 0.13) with change in MM was absent. Similarly, no correlation was found between caloric debt (r = -0.057, p = 0.631) and protein debt (r = -0.095, p = 0.424) with changes in PA.

**Conclusions:**

bioimpedance analysis demonstrated that muscle mass and phase angle were significantly lower after 7 days in ICU. The total amount of calories and proteins does not correlate with changes in muscle mass and phase angle.

## Background

Critically ill patients experience acute organ damages and disturbed endocrine and metabolic homeostasis [1]. Activation of the inflammatory response and associated endocrine changes drive hypercatabolism to mobilize substrates for energy production rapidly [2, 3]. In the acute phase of critical illness, muscle proteins form a significant source of energy because muscle proteolysis delivers amino acids that are subsequently used for gluconeogenesis [4]. As a result, skeletal muscle mass rapidly reduces [5]. The severity of muscle wasting is closely associated with illness severity [6, 7]. Muscle wasting is one of the key drivers of ICU acquired weakness (ICU-AW), which not only impairs short-term outcomes but it is also associated with impaired physical abilities and reduced quality of life 5 years after ICU discharge [8].

At the bedside, ultrasound can quantify muscle mass [9]. However, ultrasound only quantifies specific muscle groups, which may not reflect total muscle mass [10, 11]. Moreover, evaluation must be done at the same site for repeated measures to avoid bias, which requires well-trained practitioners [12, 13]. Bioimpedance analysis (BIA) could overcome these limitations, although the technique is not well validated in critically ill patients. There is limited data on the potential of BIA to assess muscle wasting in critical illness.

The primary aim of this study was to assess muscle wasting evaluated with BIA in critically ill patients at ICU admission and after 7 days. A secondary aim was to investigate if the provided energy and protein doses during the first week in ICU were associated with BIA-assessed muscle loss.

## Material and methods

### Setting and design

This is a pre-planned secondary analysis of the NUTRITI STUDY, a single-center prospective observational study that evaluated gastrointestinal dysfunction in critically ill patients [14]. The study was performed at the Department of Intensive Care of the Academic Hospital of Udine, a 1000-bed third-level acute care hospital in the North-East of Italy, and the protocol was approved by Ethics Committee of Friuli-Venezia-Giulia region (CEUR-2019-Os-17). The principles of good clinical practice performed the protocol. Written informed consent was obtained from the patient or patient representative before enrolment in the study. The study protocol was registered on ClinicalTrials.gov (NCT05473546).

All consecutive patients admitted to the general ICU from September 1st to October 30th, 2019 and from August 1st to October 30th, 2021 were evaluated for inclusion. No data were collected between the two study periods due to the COVID-19 pandemic, leading to a lack of available research staff [15].

### Inclusion and exclusion criteria

We included patients ≥ 18 years old with an expected ICU-stay ≥ 72 h and the need for artificial nutritional support, either enteral or parenteral. We excluded those with a pre-planned admission to the ICU as a consequence of elective major surgery, patients who did not require any nutritional support, those with a known gastrointestinal dysfunction (for example, gastrointestinal fistula, chronic diarrhoea or history of malabsorption); patients who tested positive for SARS-CoV2 infection (through PCR nasal swab analysis); those with a severe chronic renal disease (defined as estimated glomerular filtration rate < 30 mL/min) or liver disease (diagnosed cirrhosis and/or patient on a waiting list for liver transplantation); requiring renal replacement therapies (continuous renal replacement therapy [CRRT] or hemodialysis [HD]); patients with body mass index (BMI) < 16 kg/m^2^ or > 40 kg/m^2^; patients with neuromuscular disease; patients without BIA parameters recorded; lastly, patients who refused to undergo BIA evaluation.

### Recorded data

The following patient’s information was collected: (1) general variables (age, sex, weight, height [supine length measurement: using a flexible measuring tape the length between the vertex of the head and the heel was measured], BMI, admission diagnosis, APACHE II [Acute Physiologic Assessment and Chronic Health Evaluation] score, SOFA [Sequential Organ Failure Assessment] score; (2) clinical variables: mechanical ventilation duration, vasopressor use (continuous infusion for at least 24 h), length of ICU and hospital stay (ICU_LOS_ and HOSP_LOS_ respectively), mortality at 30–90-180 days; (3) nutritional variables (NUTRIC [Nutrition Risk in Critically ill] score at ICU admission, serum albumin-white blood cell-haemoglobin-creatinine at ICU admission, cumulative fluid balance after first ICU week (including perspiratio insensibilis calculated as following [intubated patients 0.35 mL/Kg/h, adding 0.1 mL/Kg/h for every 1 °C above 38 °C; non intubated patients 0.5 mL/Kg/h]; 4) BIA analysis data (resistance [Rz], reactance [Xc], muscle mass [MM], extracellular body water [ECW], phase angle [PA]) at ICU admission and after 7 days.

Data were pseudonymized and collected daily into a dedicated Microsoft Excel^®^ sheet (v. 2019, Redmond, WA).

Data were reported according to the STrengthening the Reporting of OBservational studies in Epidemiology (STROBE) statements to improve truthfulness and guarantee clarity.

### Bioelectrical impedance analysis

Whole-body impedance data were obtained using a tetrapolar impedance plethysmography (EFG V.3 Akern, Florence, Italy) at ICU admission and at day 7. The bioelectrical parameters of resistance (R) and reactance (Xc) were measured using an electric alternating current flux of 400 μA and an operating frequency of 50 kHz.

In brief, BIA evaluates some characteristics of tissues in response to an application of alternate current [16]. Electrolyte-rich tissues are highly conductive to electrical current, while anhydrous tissues (like fat) resist the current flow. The opposition to the flow of a current is called resistance (R), while the opposition to a current change due to a capacitor is defined as reactance (X_c_). The total opposition to an electrical current by both resistance and reactance is the impedance dimension (I) [17]. Single frequency BIA measures only extracellular water (ECW). Considering the participant’s height, age and gender in the regression equations, BIA can estimate lean body mass from impedance values and body water content [18].

Moreover, BIA provides PA, which derives from a phase shift caused by resistance to flow determined by capacitors (i.e., healthy cell membranes) that delay the current’s flow [19]. High PA correlates with large quantities of intact cell membranes and body cell mass.

Whole-body impedance measurements were taken according to the standard protocols available [20]. After the patient had remained in a supine position with arms separated from trunk by about 30° and legs separated by about 45° for at least 5 min and after the skin was cleaned to ensure good contact, appropriate electrodes were attached to the right hand and foot, according to the standard protocol of the National Institutes of Health technology assessment conference statements [21]. Enteral nutrition was stopped at least 2 h before measurement.

### Nutritional protocol, calories and protein debt calculation

As per our internal protocol and according to ESPEN Guidelines [22], our total energy and protein target is 20 kcal/kg and 1.3 g/kg/day after the first week in the ICU. Unless contraindicated, we aim to reach at least 70% of this target at day 4 after ICU admission. Continuous enteral nutrition is the preferred way to achieve this goal, while parenteral nutrition is considered when nutritional targets are not reached after 7 days.

To enable calculation of an energy and protein deficit, the energy and protein target during the first 7 days was defined as follows: 20% of 20 kcal/kg and of 1.3 g/kg actual body weight on the first day, 30% the second day, 50% at day 3, and at least 70% from day 4 to 7. The difference between the prescribed dose of total calories (Kcal_TAR_) and proteins (Prot_TAR_), and the adequate nutritional amount delivered (Kcal_EFF_ and Prot_EFF_) considering the 7 days together, has been called caloric (Kcal_DEBT_) and protein (Prot_DEBT_) deficit respectively according to the following formulas: Kcal_DEBT_ = Kcal_TAR_ − Kcal_EFF_ and Prot_DEBT_ = Prot_TAR_ − Prot_EFF_.

### Primary aim

The primary aim of this study was to measure muscle mass changes evaluated through BIA after 7 days of ICU stay.

### Secondary aims

Secondary aims were to investigate if the muscular mass estimated with BIA correlates with -BIA-derived PA, and to investigate the correlation between both Kcal_DEBT_ and Prot_DEBT_ and the change in muscle mass and PA as assessed by BIA.

### Statistical analysis

Continuous variables are reported as median (interquartile range [IQR]) or mean (SD) as appropriate, and categorical variables are reported as frequencies and percentages. According to the nature of the variables, differences between groups were assessed via t-test, Chi-squared test, and Fisher’s exact test. The normality of the distribution was evaluated by the Shapiro–Wilk test.

Spearman or Pearson’s test studied the correlation between variables as appropriate. Data were analysed using R software (The R Foundation), while graphics were made with GraphPad Prism (Version 10.0.39).

A p < 0.05 was statistically significant.

## Results

### Patient characteristics

**Fig. 1:**
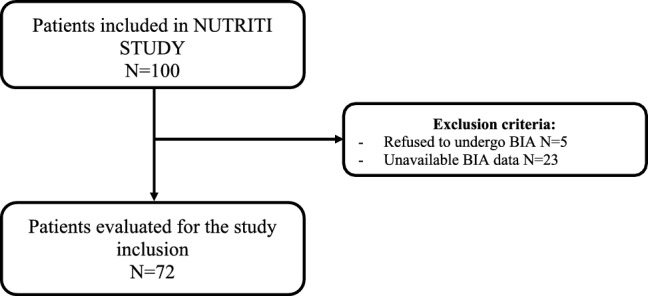
Study flowchart

During the study period, 72 patients were included as shown in Fig. 1.

Median age was 68 [59–77] years, mainly men (72%) admitted with respiratory failure (25%) (Table 1). All patients required invasive mechanical ventilation for 7 [4–10] days, and 83% of patients received vasopressor support (Table 2). Median ICU_LOS_ and HOSP_LOS_ was 8 [6–12] and 17 [11–30] days respectively (Table 2). Mortality at 180-days was 28%.

**Table 1:** Patient characteristics at ICU admission

	Overall (N = 72)
General variables	
Age (years)	68 [59–77]
Sex (male)	52 (72%)
Height (m)	1.75 [1.65–1.80]
Weight (Kg)	81 [69–90]
Admission BMI (Kg/m^2^)	27 [24–29]
Reason of admission in ICU	
Respiratory failure	18 (25%)
Trauma	13 (18%)
Neurological	14 (19%)
Emergency surgery	10 (14%)
Cardiovascular	7 (10%)
Sepsis*	4 (6%)
Other	6 (8%)
APACHE II score	20 [16–25]
SOFA score	7 [6–8]
NUTRIC score	5 [4–6]
Albumin (mg/dL)	29 [18, 27–36]
WBC 10^3/mm^3^	10.26 [8.47–13.36]
Hb (g/dL)	10.3 [9.7–11.72]
Creatinine (mg/dL)	1.22 [0.92–1.65]

BMI, Body Mass Index; ICU, Intensive Care Unit; APACHE II, Acute Physiologic Assessment and Chronic Health Evaluation II; SOFA, Sequential Organ Failure Assessment; NUTRIC, Nutrition Risk in Critically ill; WBC, White Blood Cells

^*^Sepsis from non-respiratory cause

**Table 2:** Clinical outcomes

Clinical outcomes	
MV duration (days)	7 [4–10]
Vasopressors use (%)	60 (83%)
Cumulative fluid balance at day 7 (mL)	− 1601 [− 3871—+ 876]
MV duration (days)	
ICU_LOS_	8 [6–12]
HOSP_LOS_	17 [11–30]
Mortality at 30-days	14 (19%)
Mortality at 90-days	16 (22%)
Mortality at 180-days	20 (28%)

MV, Mechanical Ventilation; ICU, Intensive Care Unit; LOS, Length of stay

### Changes in BIA-assessed muscle mass

#### BIA data were obtained after 6 [3–11] hours after admission

**Fig. 2:**
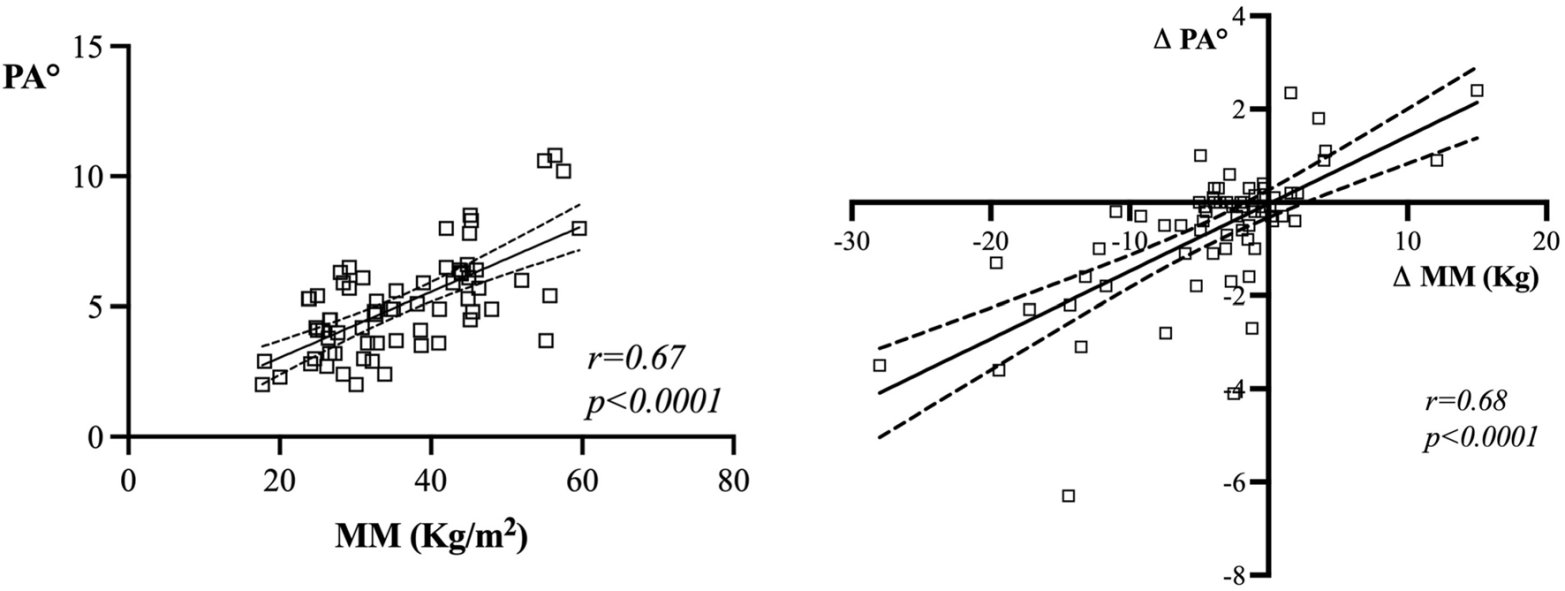
Correlations between MM and PA at ICU admission and their changes after 7 days of ICU stay. In the left part of the figure, it is shown the correlation between MM and PA at ICU admission (r = 0.67, p < 0.0001): In the right part, MM and PA changes after 7 days from admission demonstrated still to be correlated significantly (r = 0.68, p < 0.0001)

BIA at ICU admission and after 7 days showed that MM and PA significantly reduced after 7 days of critically illness (Table 3). Median MM loss was 2.65 [0.40–5.15] Kg, accounting for 8.4% [1–14] MM reduction after 7 days of ICU stay. A positive correlation was found between MM and PA at admission (r = 0.67, p < 0.0001) as shown in Fig. 2 (left part).

**Table 3:** BIA parameters changes after 7 days of ICU stay

	ICU admission	DAY 7	p
Rz (Ω)	412 [354–504]	409 [351–458]	0.0096
Xc (Ω)	36 [18, 26–45]	32 [18, 23–41]	0.0079
ECW (L)	24.2 [20.2–26.5]	23.9 [20.5–29.1]	0.3463
MM (Kg)	34.3 [27.4–59.6]	30.6 [18, 27–37]	< 0.0001
PA (°)	4.90 [3.65–6.07]	4.35 [3.32–5.47]	0.0004

Rz, resistance; Xc, reactance; ECW, extracellular water; MM, muscle mass; PA, phase angle.

Similarly, changes in MM correlated with changes in PA after 7 days in ICU (r = 0.68, p < 0.0001), see Fig. 2 (right part).

At ICU admission, there was no significant difference in MM nor PA values between patients who subsequently survived versus died during the 180-day follow up period, 32.8 [26.8–44.7] vs 35.2 [28–44.8] Kg (p = 0.44) and 4.8° [3.6–6] vs 5.4° [3.7–6] respectively (p = 0.58). However, a trend toward higher MM loss was recorded in 180-day non-survivors than in surviving patients, 2.95 kg [1.42–11.53] vs 1.85 kg [0.20–4.67] respectively, p = 0.058. Likewise, patients not surviving until 180 days after admission had a greater PA reduction after 7 days of ICU stay than survivors (− 0.70° [− 1.78–0.1] in non-survivors vs -0.15° [− 0.65–0.17] in survivors, p = 0.048).

### Association of a nutritional deficit with BIA-derived changes in muscle mass and phase angle

**Fig. 3:**
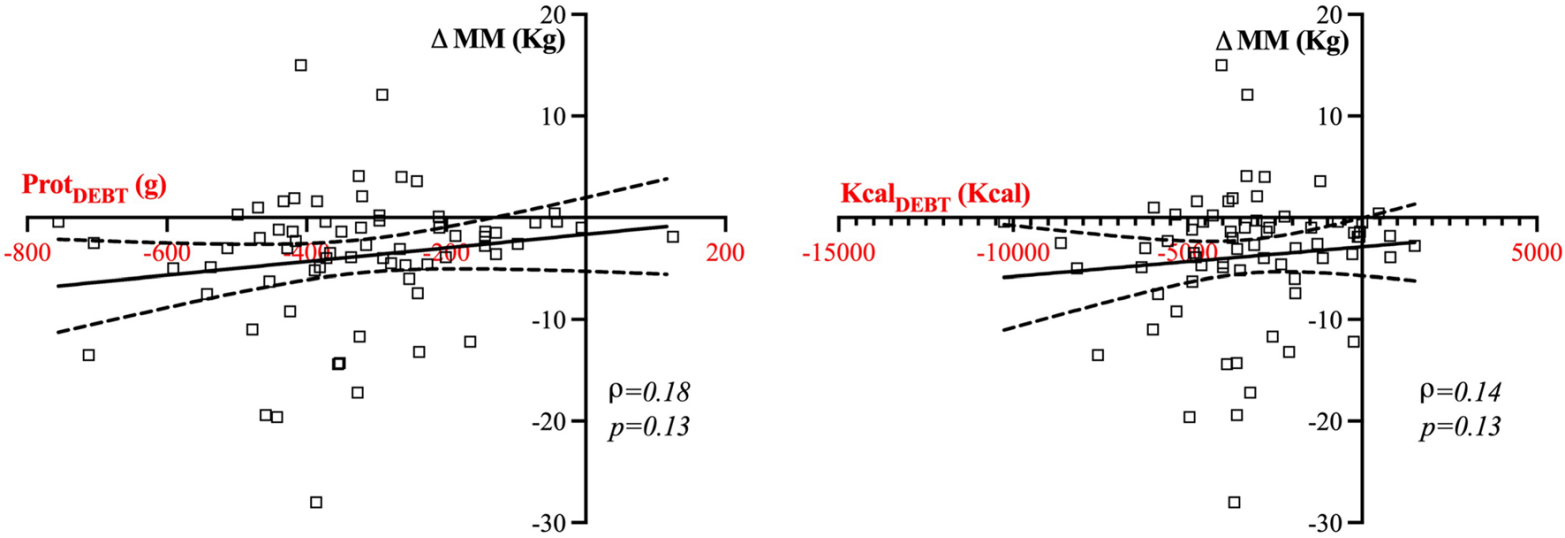
Correlation between Kcal_DEBT_ and Prot_DEBT_, and changes in MM after 7 days of ICU stay. No significant correlation was found between variations in MM (ΔMM) and protein debt (Prot_DEBT_) or caloric debt (Kcal_DEBT_) during the first 7 days after ICU admission (p = 0.13 for both correlations)

The median Kcal_TAR_ to deliver during the first 7 days was 6642 kcal [5740–7380] and the median Prot_TAR_ was 494.9 g [427.7–549.9]. Median Kcal_EFF_ provided during the first 7 days was 3271 kcal [2145–4818] and median Prot_EFF_ was 147.2 g [90.4–256.5], determining a median Kcal_DEBT_ of 3340 kcal [1574–4763] and Prot_DEBT_ of 326 g [214.8–426.7]. There was no correlation between both Kcal_DEBT_ (ρ = 0.14, p = 0.13) and Prot_DEBT_ (ρ = 0.18, p = 0.13), and changes in MM over the first week (Fig. 3).

**Fig. 4:**
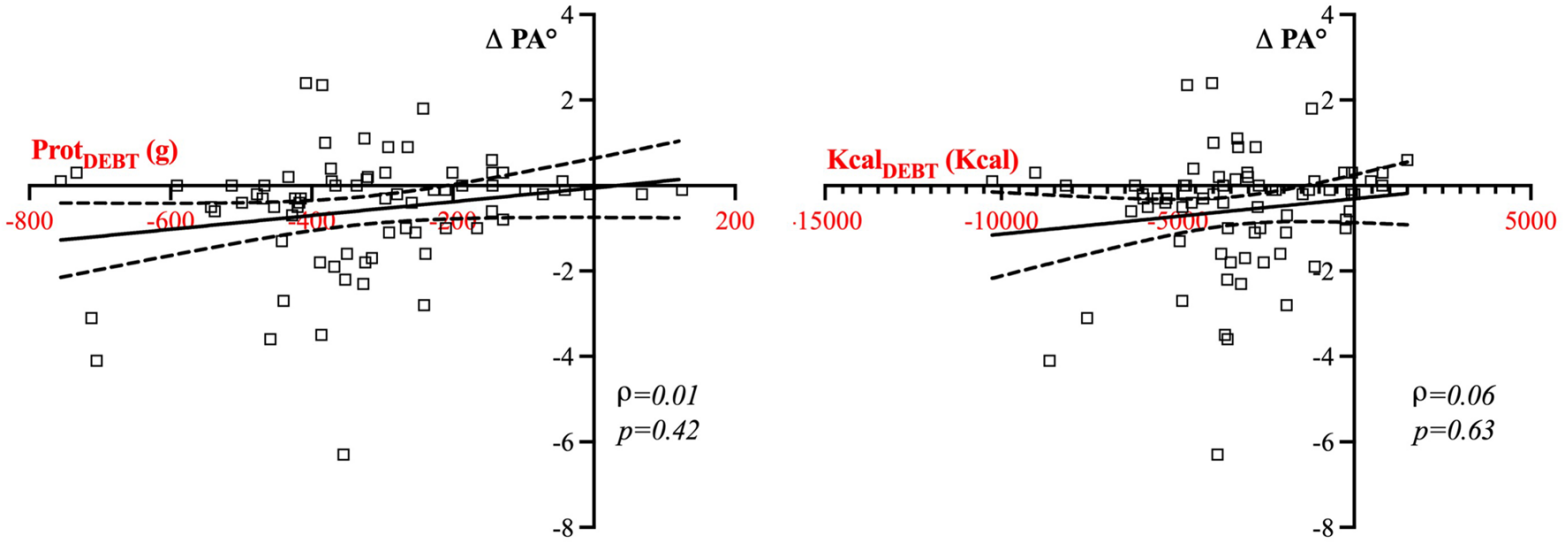
Correlation between Kcal_DEBT_ and Prot_DEBT_, and changes in PA after 7 days of ICU stay. No significant correlation was found between variations in PA (ΔPA) and protein debt (Prot_DEBT_) or caloric debt (Kcal_DEBT_) during the first 7 days after ICU admission (p = 0.42 and p = 0.63 respectively)

Similarly, there was no correlation between both Kcal_DEBT_ (ρ = -0.06, p = 0.63) and Prot_DEBT_ (ρ = − 0.09, p = 0.42), and changes in PA (Fig. 4).

## Discussion

In this observational study in long-stay critically ill patients, we found that muscle mass assessed by BIA was significantly reduced after 7 days in ICU, with more severe muscle mass loss in patients not surviving the 180-days follow-up period. Muscle mass and changes in muscle mass correlated with BIA-derived phase angle and changes in phase angle respectively. Hence, changes in phase angle could result from changes in muscle mass in critical illness. An early energy and protein deficit did not correlate with changes in muscle mass.

Preventing muscle weakness is important in critical care medicine, as ICU-acquired weakness is associated with physical impairment and reduced quality of life that may persist for a long time after ICU admission [23, 24]. ICU-acquired weakness is provoked by critical illness polyneuropathy, critical illness myopathy, and loss of muscle mass. In our study we found an 8.4% reduction of MM after 7 days of ICU stay, corresponding to a net 3.84 kg MM loss, which is quite substantial. Although non-survivors had similar muscle mass upon ICU admission, they tended to have a more significant early muscle mass loss than surviving patients, confirming the functional relevance of early muscle mass loss.

Our findings are in line with previous studies that documented substantial early mass loss in critical illness, especially in patients with the high illness severity [25]. Indeed, Puthucheary et al. reported a 12.5% reduction of rectus femoris cross-sectional area after 7 days measured by ultrasound, with increased muscle loss in patients with more severe organ dysfunction [6]. In a meta-analysis, Fazzini et al. reported approximately 2% muscle mass loss per ICU Day in the first week [26]. However, preceding studies primarily evaluated muscular mass changes by ultrasound and computed tomography (CT). Ultrasound only allows evaluation of specific muscle groups, and interrater variability may be high [10]. A CT scan cannot be performed at the bedside and exposes the patient to radiation. Theoretically, these drawbacks of ultrasound and CT could be avoided by BIA assessment of muscle mass [26]. However, the applicability of BIA in critically ill patients has been debated, since increased tissue edema may be a confounding factor [27, 28]. Nevertheless, recent studies suggest the potential usefulness of BIA in critical illness to assess muscle mass. Indeed, 2 studies, altogether including 245 critically ill patients, found a good correlation between BIA-assessed muscle mass and CT findings [29, 30].

The phase angle, a parameter derived from the arc-tangent value of the ratio of reactance to resistance, is independent of conventional regression equations and it is correlated with muscle quantity and quality [18, 31].

In the present work we found a significant correlation between PA and BIA derived MM at ICU admission (r = 0.67, p < 0.0001). Further, the correlation between absolute variations of PA and MM during the first week of ICU stay was still present (r = 0.68, p < 0.0001).

According to the results and the literature, PA significantly decreased after one week in ICU stay with a concomitant reduction in muscle mass [32].

Therefore, monitoring PA variations may be a simple tool to evaluate MM variations in ICU.

The absent correlation between early muscle wasting and a protein and energy deficit confirms anabolic resistance in the acute phase of critical illness. Indeed, despite observational studies associating a higher protein intake with improved outcomes [33], recent large randomized controlled trials have shown potential harm by increased nutritional intake and increased protein doses in the acute phase of critical illness [34–37]. Indeed, the EPaNIC trial showed harm by early parenteral nutrition supplementing insufficient enteral nutrition compared with withholding parenteral nutrition until one week after ICU admission [35]. Likewise, the NUTRIREA-3 trial showed harm by early complete nutrition compared to restrictive feeding through any route [36]. In both RCTs, urea levels increased in the group receiving extra amino acids, suggesting futile catabolism of the supplementary provided amino acids [38]. Likewise, in a secondary analysis of patients included in the EPaNIC study, CT-assessed muscle mass loss was not prevented by early parenteral nutrition [39], and the incidence of weakness even increased [40]. Harm in the EPaNIC trial was subsequently attributed to the increased protein doses, and to feeding-induced suppression of autophagy, a crucial cellular recovery process [40–42]. Similarly, in the recent post-hoc analysis of the EFFORT trial, increased protein doses did not improve outcome of critically ill patients, and the intervention increased ureagenesis and associated with increased mortality in patients with acute kidney injury and high organ failure scores [43]. The time point when anabolic responsiveness switches into feeding responsiveness remains to be defined and likely varies between patients. BIA may assist in documenting the presence or absence of an anabolic response to artificial nutrition in intervention studies, which requires further study.

Contrary to Thibault et al. [44], we did not observe a significant ICU admission phase angle difference between survivors and non-survivors at 28-day follow-up. However, this study found that patients not surviving until 180 days after admission had a greater PA reduction than survivors (p = 0.048). This may reflect the effect of a higher disease severity, which in turn leads to the patient's death. Moreover, we cannot exclude that the greater PA decrease in non-survivors could result from a maladaptive host response to the acute illness, such as recently shown in critically ill COVID-19 patients [45].

Some limitations should be acknowledged. Firstly, the study is observational, and we did not correct for potential confounders. However, recent randomized controlled trials confirm anabolic resistance in the acute phase of critical illness.

Secondly, we did not assess other markers of muscle mass or function. Future studies should investigate the accuracy and agreement in low muscle mass identification using diverse markers compared to changes in BIA-assessed muscle mass [46]. However, the degree of early muscle mass loss was similar to studies assessing muscle wasting by other techniques, and the more significant muscle wasting in non-surviving patients compared to surviving patients supports the functional relevance of our findings.

Third, we did not evaluate the fluid intake from ICU admission to the obtainment of BIA values, which could have altered PA or MM estimation. However, median time from ICU admission to available BIA data was short, and near 50% of patients had an acute illness that did not require large fluidic volume resuscitation.

Lastly, we included a low proportion of septic patients considered at higher risk of muscle derangements than another category of ICU patients [47].

## Conclusions

In conclusion, BIA can provide potentially helpful information on muscle wasting in the ICU, and PA may be a valid substitute for MM in the ICU. An early protein and energy deficit did not correlate with increased muscle wasting, supporting the concept of anabolic resistance in the acute phase of critical illness.

## Data Availability

The datasets generated and/or analysed during the current study are not publicly available due privacy concerns, but are available from the corresponding author on reasonable request.

## Abbreviations

BIA: Bioimpedance analysis
ICU: Intensive care unit
MM: Muscular mass
PA: Phase angle
ICU-AW: ICU acquired weakness
CRRT: Continuous renal replacement therapy
HD: Hemodialysis
BMI: Body mass index
APACHE: Acute Physiologic Assessment and Chronic Health Evaluation score
SOFA: Sequential Organ Failure Assessment score
ICU_LOS_: Length of ICU stay
HOSP_LOS_: Length of hospital stay
NUTRIC: Nutrition Risk in Critically ill
Rz: Resistance,
Xc: Reactance
ECW: Extracellular body water
